# 5,5,7,12,12,14-Hexamethyl-1,8-bis­(4-nitro­benz­yl)-1,4,8,11-tetra­aza­cyclo­tetra­deca­ne

**DOI:** 10.1107/S1600536813031164

**Published:** 2013-12-14

**Authors:** K. Gayathri, S. Sathya, G. Usha, G. Ramanjaneya Reddy, S. Balasubramanian

**Affiliations:** a PG and Research Department of Physics, Queen Mary’s College, Chennai-4, Tamilnadu, India; b Department of Inorganic Chemistry, University of Madras, Maraimalai Campus, Chennai-25, Tamilnadu, India

## Abstract

The asymmetric unit of the title compound, C_30_H_46_N_6_O_4_, contains one half-mol­ecule. The C(benzene)—C(CH_2_)—N—C(—Me) torsion angle is −79.89 (13)° suggesting a synclinal orientation of the nitro­benzene ring with respect to the macrocycle. The conformation of the macrocycle is stabilized by intra­molecular N—H⋯N hydrogen bonds.

## Related literature   

For the biological activity of cyclam derivatives, see: Cronin *et al.* (1999 ▶); Fzerov *et al.* (2005 ▶). For related structures, see: Xie *et al.* (2008 ▶); Feng *et al.* (2009 ▶). 
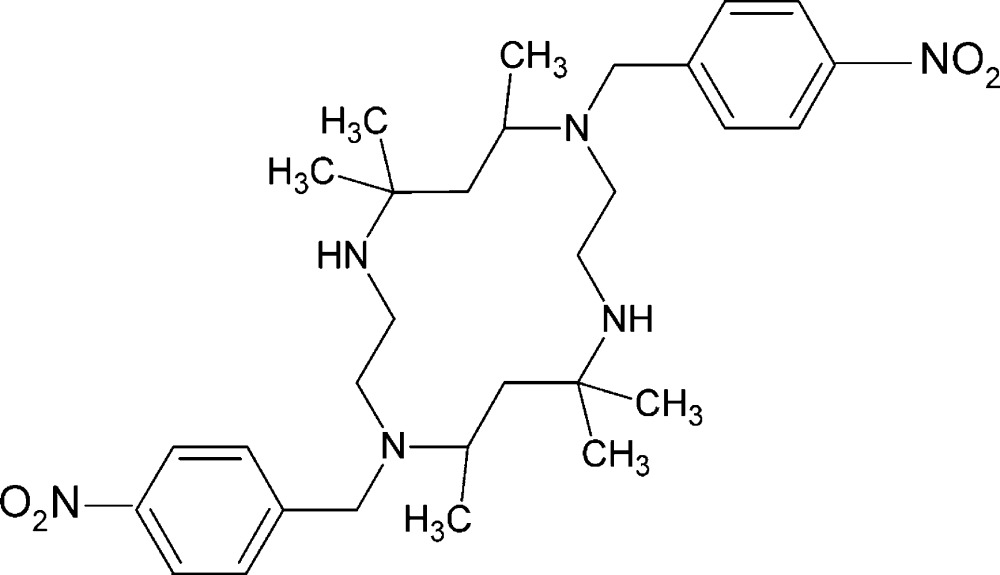


## Experimental   

### 

#### Crystal data  

C_30_H_46_N_6_O_4_
*M* *_r_* = 554.73
Triclinic, 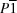

*a* = 8.6407 (4) Å
*b* = 9.1433 (3) Å
*c* = 11.0008 (5) Å
α = 107.742 (2)°
β = 104.898 (2)°
γ = 102.372 (2)°
*V* = 758.45 (6) Å^3^
*Z* = 1
Mo *K*α radiation
μ = 0.08 mm^−1^
*T* = 273 K
0.35 × 0.30 × 0.25 mm

#### Data collection  

Bruker Kappa APEXII CCD diffractometer
Absorption correction: multi-scan (*SADABS*; Bruker, 2004 ▶) *T*_min_ = 0.972, *T*_max_ = 0.980
18201 measured reflections
4819 independent reflections
3340 reflections with *I* > 2σ(*I*)
*R*_int_ = 0.028

#### Refinement  

*R*[*F* ^2^ > 2σ(*F* ^2^)] = 0.051
*wR*(*F* ^2^) = 0.185
*S* = 0.96
4819 reflections
185 parameters
H atoms treated by a mixture of independent and constrained refinement
Δρ_max_ = 0.22 e Å^−3^
Δρ_min_ = −0.22 e Å^−3^

### 

Data collection: *APEX2* (Bruker, 2004 ▶); cell refinement: *APEX2* and *SAINT* (Bruker, 2004 ▶); data reduction: *SAINT* and *XPREP* (Bruker, 2004 ▶); program(s) used to solve structure: *SIR92* (Altomare *et al.*, 1993 ▶); program(s) used to refine structure: *SHELXL97* (Sheldrick, 2008 ▶); molecular graphics: *OLEX2* (Dolomanov *et al.*, 2009 ▶); software used to prepare material for publication: *SHELXL97*.

## Supplementary Material

Crystal structure: contains datablock(s) I, New_Global_Publ_Block. DOI: 10.1107/S1600536813031164/vm2199sup1.cif

Structure factors: contains datablock(s) I. DOI: 10.1107/S1600536813031164/vm2199Isup2.hkl

Supporting information file. DOI: 10.1107/S1600536813031164/vm2199Isup3.cml

Additional supporting information:  crystallographic information; 3D view; checkCIF report

## Figures and Tables

**Table 1:** Hydrogen-bond geometry (Å, °)

*D*—H⋯*A*	*D*—H	H⋯*A*	*D*⋯*A*	*D*—H⋯*A*
N3—H3*A*⋯N2^i^	0.883 (17)	2.284 (17)	2.9770 (15)	135.3 (15)

Symmetry code: (i) 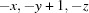.

**Fig. 1.**
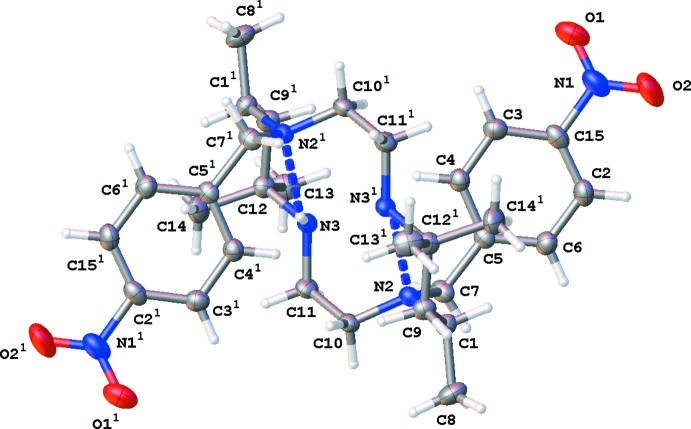
The molecular structure of the title compound, with displacement ellipsoids drawn at the 30% probability level and hydrogen bonds shown as broken lines. [Symmetry code: (1) -x, -y + 1, -z]

## supplementary crystallographic information

### 1. Comment

Cyclam based complexes have been used in a wide range of studies from bioinorganic systems to catalytic systems and as sensors (Cronin *et al.*, 1999). Cyclam based anti-HIV agents are more active *in vivo* in the form of metal ion complexes. Macrocyclic ligands are also commonly used as carriers of metal radioisotopes in targeted radiopharmaceuticals. For utilization in nuclear medicine, macrocyclic ligands are generally preferred to open-chain ligands due to the higher thermodynamic and mainly kinetic stabilities of their complexes (Fzerov *et al.*, 2005).

As part of our studies to examine the cyclam derivatives,we report the structure of the title compound (Fig. 1). The C–C bond lengths of the methyl groups attached to the macrocycle [C12—C13 = 1.535 (2) Å, C12—C14 = 1.532 (2) Å and C1—C8 = 1.533 (3) Å] are in good agreement with the literature values [C6—C7=1.53 (5) Å, C6—C8=1.541 (5) Å and C3—C5=1.535 (4) Å in Xie *et al.*, 2008]. The bond angle C11—N3—C12 in the cyclam ring is 115.96 (1)° which agrees with the value 115.4 (3)° of the related reported structure (Feng *et al.*, 2009). The sum of the angles around N1 atom [360 °], N2 atom [339.44°] and N3 atom [333.76 °] is an indication of *sp*^2^, *sp*^3^ and *sp*^3^hybridization, respectively. The conformation of the macrocycle is stabilized by intramolecular N—H···N hydrogen bonds (Table 1).

### 2. Experimental

The ligand 1,8-bi(*para*-nitro benzyl)-5,5,7,12,12,14-hexamethyl-1,4,8,11-tetraazacyclotetradecane (*L*) (0.57 g, 2 mmol) was dissolved in 20 ml of methanol, and then sodium carbonate (0.636 g, 6 mmol) dissolved in 2 ml of water and potassium iodide (1 g, 6 mmol) were added to the above solution. Para-nitrobenzyl bromide (0.95 g, 4.4 mmol) in methanol was slowly added to the reaction mixture and refluxed for 12 h. The resulting yellow color product was washed with water, methanol and diethyl ether and it was recrystallized from a mixture of chloroform-methanol (75:25). Yield 0.83 g (75%).

### 3. Refinement

H atoms were positioned geometrically and treated as riding on their parent atoms, with C—H distances of 0.93–0.98 Å and *U*_iso_(H)= 1.5*U*_eq_(*C*-methyl) or *U*_iso_(H)= 1.2*U*_eq_(N,*C*) for other H atoms. H3A was found in a difference Fourier map.

### Crystal data

**Table:**

C_30_H_46_N_6_O_4_	*V* = 758.45 (6) Å^3^
*M**_r_* = 554.73	*Z* = 1
Triclinic, *P*1	*F*(000) = 300
Hall symbol: -P 1	*D*_x_ = 1.215 Mg m^−^^3^
*a* = 8.6407 (4) Å	Mo *K*α radiation, λ = 0.71073 Å
*b* = 9.1433 (3) Å	θ = 1.0–31.1°
*c* = 11.0008 (5) Å	µ = 0.08 mm^−^^1^
α = 107.742 (2)°	*T* = 273 K
β = 104.898 (2)°	Block, colourless
γ = 102.372 (2)°	0.35 × 0.30 × 0.25 mm

### Data collection

**Table:**

Bruker Kappa APEXII CCD diffractometer	4819 independent reflections
Radiation source: fine-focus sealed tube	3340 reflections with *I* > 2σ(*I*)
Graphite monochromator	*R*_int_ = 0.028
ω and φ scan	θ_max_ = 31.1°, θ_min_ = 2.1°
Absorption correction: multi-scan (*SADABS*; Bruker, 2004)	*h* = −12→12
*T*_min_ = 0.972, *T*_max_ = 0.980	*k* = −13→11
18201 measured reflections	*l* = −15→15

### Refinement

**Table:**

Refinement on *F*^2^	Primary atom site location: structure-invariant direct methods
Least-squares matrix: full	Secondary atom site location: difference Fourier map
*R*[*F*^2^ > 2σ(*F*^2^)] = 0.051	Hydrogen site location: inferred from neighbouring sites
*wR*(*F*^2^) = 0.185	H atoms treated by a mixture of independent and constrained refinement
*S* = 0.96	*w* = 1/[σ^2^(*F*_o_^2^) + (0.1167*P*)^2^ + 0.0862*P*] where *P* = (*F*_o_^2^ + 2*F*_c_^2^)/3
4819 reflections	(Δ/σ)_max_ < 0.001
185 parameters	Δρ_max_ = 0.22 e Å^−^^3^
0 restraints	Δρ_min_ = −0.22 e Å^−^^3^

### Special details

**Table:**

Geometry. All e.s.d.'s (except the e.s.d. in the dihedral angle between two l.s. planes) are estimated using the full covariance matrix. The cell e.s.d.'s are taken into account individually in the estimation of e.s.d.'s in distances, angles and torsion angles; correlations between e.s.d.'s in cell parameters are only used when they are defined by crystal symmetry. An approximate (isotropic) treatment of cell e.s.d.'s is used for estimating e.s.d.'s involving l.s. planes.
Refinement. Refinement of *F*^2^ against ALL reflections. The weighted *R*-factor *wR* and goodness of fit *S* are based on *F*^2^, conventional *R*-factors *R* are based on *F*, with *F* set to zero for negative *F*^2^. The threshold expression of *F*^2^ > σ(*F*^2^) is used only for calculating *R*-factors(gt) *etc*. and is not relevant to the choice of reflections for refinement. *R*-factors based on *F*^2^ are statistically about twice as large as those based on *F*, and *R*- factors based on ALL data will be even larger.

### Fractional atomic coordinates and isotropic or equivalent isotropic displacement parameters (Å^2^)

**Table:**

	*x*	*y*	*z*	*U*_iso_*/*U*_eq_	
C1	0.17573 (15)	0.33128 (15)	0.19940 (12)	0.0405 (3)	
H1	0.1959	0.3975	0.2942	0.049*	
C2	0.26370 (18)	0.93648 (14)	0.50435 (14)	0.0469 (3)	
C3	0.19243 (17)	0.89974 (15)	0.36661 (14)	0.0466 (3)	
H3	0.1203	0.9525	0.3346	0.056*	
C4	0.23154 (16)	0.78245 (15)	0.27817 (13)	0.0431 (3)	
H4	0.1849	0.7554	0.1850	0.052*	
C5	0.33941 (14)	0.70422 (13)	0.32604 (12)	0.0384 (3)	
C6	0.40708 (17)	0.74400 (16)	0.46470 (13)	0.0469 (3)	
H6	0.4791	0.6915	0.4974	0.056*	
C7	0.37823 (15)	0.57761 (15)	0.22457 (13)	0.0441 (3)	
H7A	0.4292	0.6259	0.1712	0.053*	
H7B	0.4584	0.5366	0.2724	0.053*	
C8	0.2822 (2)	0.2174 (2)	0.20329 (19)	0.0615 (4)	
H8A	0.3998	0.2799	0.2438	0.092*	
H8B	0.2596	0.1444	0.1123	0.092*	
H8C	0.2537	0.1565	0.2562	0.092*	
C9	−0.01195 (16)	0.23196 (14)	0.13605 (14)	0.0427 (3)	
H9A	−0.0295	0.1501	0.1746	0.051*	
H9B	−0.0366	0.1755	0.0396	0.051*	
C10	0.22706 (16)	0.36646 (14)	−0.00178 (12)	0.0395 (3)	
H10A	0.1226	0.2776	−0.0545	0.047*	
H10B	0.3190	0.3208	0.0056	0.047*	
C11	0.24794 (16)	0.47711 (16)	−0.07869 (14)	0.0439 (3)	
H11A	0.3646	0.5463	−0.0422	0.053*	
H11B	0.2231	0.4118	−0.1735	0.053*	
C12	0.14282 (15)	0.68065 (14)	−0.15082 (13)	0.0403 (3)	
C13	0.31661 (18)	0.81153 (19)	−0.08704 (18)	0.0604 (4)	
H13A	0.4025	0.7614	−0.0944	0.091*	
H13B	0.3368	0.8705	0.0072	0.091*	
H13C	0.3193	0.8845	−0.1339	0.091*	
C14	0.1105 (2)	0.5867 (2)	−0.30121 (15)	0.0606 (4)	
H14A	0.0017	0.5051	−0.3412	0.091*	
H14B	0.1962	0.5363	−0.3088	0.091*	
H14C	0.1135	0.6599	−0.3479	0.091*	
C15	0.36900 (19)	0.86074 (17)	0.55532 (13)	0.0512 (3)	
H15	0.4139	0.8870	0.6485	0.061*	
N1	0.2281 (2)	1.06532 (16)	0.59985 (15)	0.0653 (4)	
N2	0.22416 (11)	0.44428 (11)	0.13438 (9)	0.0347 (2)	
N3	0.13852 (12)	0.57798 (12)	−0.07040 (10)	0.0348 (2)	
O1	0.1657 (2)	1.15487 (16)	0.55712 (16)	0.0867 (4)	
O2	0.2633 (3)	1.0778 (2)	0.71694 (15)	0.1117 (6)	
H3A	0.0333 (19)	0.5159 (18)	−0.0954 (14)	0.044 (4)*	

### Atomic displacement parameters (Å^2^)

**Table:**

	*U*^11^	*U*^22^	*U*^33^	*U*^12^	*U*^13^	*U*^23^
C1	0.0390 (6)	0.0426 (6)	0.0402 (6)	0.0128 (5)	0.0113 (5)	0.0180 (5)
C2	0.0518 (7)	0.0350 (6)	0.0468 (7)	0.0052 (5)	0.0239 (6)	0.0065 (5)
C3	0.0470 (7)	0.0391 (6)	0.0524 (7)	0.0137 (5)	0.0168 (6)	0.0159 (5)
C4	0.0444 (7)	0.0404 (6)	0.0378 (6)	0.0096 (5)	0.0113 (5)	0.0112 (5)
C5	0.0322 (5)	0.0337 (5)	0.0386 (6)	0.0031 (4)	0.0095 (4)	0.0070 (4)
C6	0.0462 (7)	0.0452 (6)	0.0417 (6)	0.0120 (5)	0.0087 (5)	0.0134 (5)
C7	0.0316 (5)	0.0425 (6)	0.0463 (7)	0.0077 (5)	0.0113 (5)	0.0059 (5)
C8	0.0553 (9)	0.0637 (9)	0.0778 (11)	0.0282 (7)	0.0172 (8)	0.0418 (8)
C9	0.0419 (6)	0.0371 (5)	0.0501 (7)	0.0102 (5)	0.0157 (5)	0.0198 (5)
C10	0.0407 (6)	0.0396 (6)	0.0391 (6)	0.0187 (5)	0.0156 (5)	0.0107 (5)
C11	0.0428 (6)	0.0531 (7)	0.0473 (7)	0.0237 (5)	0.0248 (5)	0.0209 (5)
C12	0.0388 (6)	0.0420 (6)	0.0438 (6)	0.0101 (5)	0.0189 (5)	0.0194 (5)
C13	0.0415 (7)	0.0556 (8)	0.0866 (11)	0.0069 (6)	0.0256 (7)	0.0333 (8)
C14	0.0729 (10)	0.0772 (10)	0.0474 (8)	0.0301 (8)	0.0322 (7)	0.0305 (7)
C15	0.0579 (8)	0.0488 (7)	0.0362 (6)	0.0087 (6)	0.0126 (6)	0.0107 (5)
N1	0.0779 (9)	0.0465 (6)	0.0655 (8)	0.0149 (6)	0.0370 (7)	0.0067 (6)
N2	0.0322 (4)	0.0321 (4)	0.0349 (5)	0.0084 (3)	0.0110 (4)	0.0082 (3)
N3	0.0325 (5)	0.0385 (5)	0.0371 (5)	0.0123 (4)	0.0163 (4)	0.0152 (4)
O1	0.1035 (11)	0.0574 (7)	0.0994 (10)	0.0386 (7)	0.0451 (9)	0.0127 (7)
O2	0.1857 (18)	0.0998 (11)	0.0652 (9)	0.0661 (12)	0.0679 (10)	0.0175 (8)

### Geometric parameters (Å, º)

**Table:**

C1—N2	1.4742 (15)	C9—H9B	0.9700
C1—C9	1.5305 (17)	C10—N2	1.4588 (15)
C1—C8	1.5328 (19)	C10—C11	1.5145 (17)
C1—H1	0.9800	C10—H10A	0.9700
C2—C15	1.366 (2)	C10—H10B	0.9700
C2—C3	1.382 (2)	C11—N3	1.4559 (16)
C2—N1	1.4675 (18)	C11—H11A	0.9700
C3—C4	1.3781 (18)	C11—H11B	0.9700
C3—H3	0.9300	C12—N3	1.4743 (15)
C4—C5	1.3859 (18)	C12—C9^i^	1.5317 (18)
C4—H4	0.9300	C12—C14	1.5318 (19)
C5—C6	1.3831 (17)	C12—C13	1.5349 (18)
C5—C7	1.5067 (17)	C13—H13A	0.9600
C6—C15	1.383 (2)	C13—H13B	0.9600
C6—H6	0.9300	C13—H13C	0.9600
C7—N2	1.4598 (14)	C14—H14A	0.9600
C7—H7A	0.9700	C14—H14B	0.9600
C7—H7B	0.9700	C14—H14C	0.9600
C8—H8A	0.9600	C15—H15	0.9300
C8—H8B	0.9600	N1—O2	1.208 (2)
C8—H8C	0.9600	N1—O1	1.214 (2)
C9—C12^i^	1.5317 (18)	N3—H3A	0.883 (15)
C9—H9A	0.9700		
			
N2—C1—C9	112.95 (9)	C11—C10—H10A	108.6
N2—C1—C8	113.69 (11)	N2—C10—H10B	108.6
C9—C1—C8	109.55 (11)	C11—C10—H10B	108.6
N2—C1—H1	106.7	H10A—C10—H10B	107.5
C9—C1—H1	106.7	N3—C11—C10	112.80 (10)
C8—C1—H1	106.7	N3—C11—H11A	109.0
C15—C2—C3	122.80 (12)	C10—C11—H11A	109.0
C15—C2—N1	118.71 (13)	N3—C11—H11B	109.0
C3—C2—N1	118.47 (14)	C10—C11—H11B	109.0
C4—C3—C2	117.93 (13)	H11A—C11—H11B	107.8
C4—C3—H3	121.0	N3—C12—C9^i^	107.94 (9)
C2—C3—H3	121.0	N3—C12—C14	113.61 (11)
C3—C4—C5	120.98 (12)	C9^i^—C12—C14	110.55 (11)
C3—C4—H4	119.5	N3—C12—C13	108.11 (11)
C5—C4—H4	119.5	C9^i^—C12—C13	106.91 (11)
C6—C5—C4	119.18 (11)	C14—C12—C13	109.47 (11)
C6—C5—C7	122.20 (12)	C12—C13—H13A	109.5
C4—C5—C7	118.62 (11)	C12—C13—H13B	109.5
C15—C6—C5	120.86 (13)	H13A—C13—H13B	109.5
C15—C6—H6	119.6	C12—C13—H13C	109.5
C5—C6—H6	119.6	H13A—C13—H13C	109.5
N2—C7—C5	110.45 (9)	H13B—C13—H13C	109.5
N2—C7—H7A	109.6	C12—C14—H14A	109.5
C5—C7—H7A	109.6	C12—C14—H14B	109.5
N2—C7—H7B	109.6	H14A—C14—H14B	109.5
C5—C7—H7B	109.6	C12—C14—H14C	109.5
H7A—C7—H7B	108.1	H14A—C14—H14C	109.5
C1—C8—H8A	109.5	H14B—C14—H14C	109.5
C1—C8—H8B	109.5	C2—C15—C6	118.25 (12)
H8A—C8—H8B	109.5	C2—C15—H15	120.9
C1—C8—H8C	109.5	C6—C15—H15	120.9
H8A—C8—H8C	109.5	O2—N1—O1	123.36 (15)
H8B—C8—H8C	109.5	O2—N1—C2	118.46 (16)
C1—C9—C12^i^	118.88 (10)	O1—N1—C2	118.18 (15)
C1—C9—H9A	107.6	C10—N2—C7	113.41 (9)
C12^i^—C9—H9A	107.6	C10—N2—C1	114.37 (9)
C1—C9—H9B	107.6	C7—N2—C1	111.66 (10)
C12^i^—C9—H9B	107.6	C11—N3—C12	115.96 (9)
H9A—C9—H9B	107.0	C11—N3—H3A	109.2 (10)
N2—C10—C11	114.82 (10)	C12—N3—H3A	108.6 (9)
N2—C10—H10A	108.6		
			
C15—C2—C3—C4	−0.6 (2)	C3—C2—N1—O2	166.54 (16)
N1—C2—C3—C4	177.77 (11)	C15—C2—N1—O1	164.41 (15)
C2—C3—C4—C5	−0.19 (19)	C3—C2—N1—O1	−14.0 (2)
C3—C4—C5—C6	0.62 (18)	C11—C10—N2—C7	−60.07 (13)
C3—C4—C5—C7	−179.70 (11)	C11—C10—N2—C1	170.32 (10)
C4—C5—C6—C15	−0.30 (19)	C5—C7—N2—C10	149.14 (10)
C7—C5—C6—C15	−179.97 (12)	C5—C7—N2—C1	−79.89 (13)
C6—C5—C7—N2	118.14 (13)	C9—C1—N2—C10	−71.93 (13)
C4—C5—C7—N2	−61.53 (15)	C8—C1—N2—C10	53.70 (14)
N2—C1—C9—C12^i^	−66.10 (14)	C9—C1—N2—C7	157.59 (10)
C8—C1—C9—C12^i^	166.07 (12)	C8—C1—N2—C7	−76.78 (13)
N2—C10—C11—N3	−45.97 (15)	C10—C11—N3—C12	−175.81 (10)
C3—C2—C15—C6	0.9 (2)	C9^i^—C12—N3—C11	176.37 (10)
N1—C2—C15—C6	−177.46 (12)	C14—C12—N3—C11	53.40 (15)
C5—C6—C15—C2	−0.4 (2)	C13—C12—N3—C11	−68.32 (14)
C15—C2—N1—O2	−15.0 (2)		

Symmetry code: (i) −*x*, −*y*+1, −*z*.

### Hydrogen-bond geometry (Å, º)

**Table:**

*D*—H···*A*	*D*—H	H···*A*	*D*···*A*	*D*—H···*A*
N3—H3*A*···N2^i^	0.883 (17)	2.284 (17)	2.9770 (15)	135.3 (15)

Symmetry code: (i) −*x*, −*y*+1, −*z*.
